# Effects of Exercise Intervention on Peripheral Skeletal Muscle in Stable Patients With COPD: A Systematic Review and Meta-Analysis

**DOI:** 10.3389/fmed.2021.766841

**Published:** 2021-11-18

**Authors:** Peijun Li, Jian Li, Yingqi Wang, Jun Xia, Xiaodan Liu

**Affiliations:** ^1^Department of Sports Rehabilitation, Shanghai University of Sport, Shanghai, China; ^2^School of Rehabilitation Science, Shanghai University of Traditional Chinese Medicine, Shanghai, China; ^3^Institute of Rehabilitation Medicine, Shanghai University of Traditional Chinese Medicine, Shanghai, China

**Keywords:** chronic obstructive pulmonary disease, exercise training, meta-analysis, skeletal muscle dysfunction, exercise capacity

## Abstract

**Objectives:** Peripheral skeletal muscle dysfunction is an important extrapulmonary manifestation of chronic obstructive pulmonary disease (COPD) that can be counteracted by exercise training. This study aimed to review the effect of three major exercise training modalities, which are used in pulmonary rehabilitation to improve on skeletal muscle mass, function, and exercise capacity in COPD.

**Methods:** PubMed, Embase, EBSCO, Web of Science, and the PEDro database were searched on April 25, 2020. Only randomized controlled studies published in English evaluating the effects of exercise interventions on peripheral skeletal muscle mass, strength, and exercise capacity in stable COPD patients were included. The quality of included studies was evaluated using the PEDro scale. The mean difference (MD) or the standardized mean difference (SMD) with 95% CI was calculated to summarize the results. Subgroup meta-analysis was used to investigate the effects of different exercise training modalities and different outcome measures. The Grading of Recommendations Assessment, Development, and Evaluation guidelines were used to rate evidence quality.

**Results:** A total of 30 randomized controlled trials involving 1,317 participants were included. Data from trials investigating endurance exercise (EE), resistance exercise (RE), and combined aerobic and resistance exercise (CE) were pooled into a meta-analysis, and the differences compared with the non-exercising COPD control were improvement in the muscle strength and exercise capacity in stable COPD patients. Subgroup meta-analysis for different exercise training modalities showed that RE significantly improved muscle strength (SMD = 0.6, 95% CI 0.35–0.84, *I*^2^ = 61%), EE and CE significantly increased VO_2peak_ (EE: MD = 3.5, 95% CI 1.1–5.91, *I*^2^ = 92%; CE: MD = 1.66, 95% CI 0.22–3.1, *I*^2^ = 1%). Subgroup meta-analysis for different outcome measures showed that only isotonic strength was improved after exercise interventions (SMD = 0.89, 95% CI 0.51–1.26, *I*^2^ = 71%).

**Conclusion:** Moderate evidence supports that exercise training in stable COPD patients has meaningful and beneficial effects on peripheral skeletal muscle strength and exercise capacity. Peripheral skeletal muscle shows a higher response to RE, and the isotonic test is relatively sensitive in reflecting muscle strength changes. The proportion of aerobic and resistance exercise components in a combined exercise program still needs exploration.

**Systematic Review Registration:** The review was registered with the PROSPERO: (The website is https://www.crd.york.ac.uk/PROSPERO/, and the ID is CRD42020164868).

## Introduction

Chronic obstructive pulmonary disease (COPD) is a common disease characterized by persistent respiratory symptoms and expiratory flow limitation (1). Furthermore, many patients with COPD experience systematic symptoms, including impaired cardiopulmonary and skeletal muscle function (2, 3). Skeletal muscle dysfunction is one of the significant systemic manifestations of COPD, characterized by the loss of muscle mass, a transition of the fiber type proportion, a decrease in the capillary to fiber ratio, and muscle strength and endurance (4, 5). In most patients with COPD, the observed decrease in muscle strength is proportional to muscle mass loss, suggesting that the onset of skeletal muscle dysfunction is caused by paralleled chronic inactivity and muscle deconditioning rather than myopathy (6). The existence of dyspnea in COPD decreases physical activity, and the decrease in physical activity induces and accelerates skeletal muscle dysfunction, worsening the dyspnea in patients, forming a vicious cycle that causes further deconditioning on COPD (7). Recently, lower limb muscle function has been associated with exercise capacity in COPD (8). Previous studies have confirmed that skeletal muscle dysfunction is an additional important contributor to COPD exercise restriction and function impairments (9, 10), and it is closely related to the quality of life, readmission rate, and mortality (11, 12).

Pulmonary rehabilitation is a comprehensive management program designed for COPD and has significant clinical effects in improving dyspnea, quality of life, and exercise capacity (1). As the cornerstone of pulmonary rehabilitation, exercise training can effectively reverse or at least stabilize the loss of skeletal muscle mass and strength in patients with COPD, and it is considered currently the most effective non-pharmaceutical intervention for COPD skeletal muscle dysfunction (13). The American Thoracic Society/European Respiratory Society (ATS/ERS) statement provided a short overview of the effects of exercise interventions on the muscle function and mass in COPD, showing that exercise interventions can improve the morphology and function of COPD skeletal muscle (12), but the included literatures are extensive and heterogeneous. Another international guideline described and analyzed the effects of different exercise modalities in COPD skeletal muscle dysfunction and provided a GRADE scale for evidence quality (4). In 2018, a review included 70 English language literature to be analyzed and concluded that exercise intervention could improve COPD skeletal muscle strength, endurance, and mass, despite the fact that intervention programs and outcome measures were heterogeneous (14). Therefore, although previous international guidelines and recent reviews have consistently concluded that exercise training improves COPD skeletal muscle dysfunction, it is still difficult to clarify the degree of real benefit due to the diversity and heterogeneity of exercise intervention programs and outcome measures. Previous meta-analysis of exercise in COPD explored the effects of resistance exercise (RE) on exercise capacity (15), endurance exercise (EE) vs. RE (16), and combined aerobic and resistance exercise (CE) vs. EE on lower limb muscle strength and exercise capacity (17). However, these studies focused on the effects of single exercise modality or the compared effects of two exercise modalities. There is still a lack of comprehensive quantitative effect of exercise on peripheral skeletal muscle mass, strength, and exercise capacity in COPD.

In this systematic review and meta-analysis, the effects of exercise interventions on peripheral skeletal muscle mass, strength, and exercise capacity in COPD were determined. The characteristics of different exercise modalities were further discussed to provide a theoretical reference for developing a targeted COPD exercise rehabilitation program.

## Methods

### Search Strategy and Selection Criteria

This systematic review and meta-analysis was registered (PROSPERO registration number: CRD42020164868) and conducted according to the preferred reporting items for systematic reviews and meta-analysis (PRISMA) recommendations (18). According to the principle of population intervention comparison outcomes, the inclusion criteria were as follows: (a) participants diagnosed with stable COPD, and without gender and age restrictions; (b) EE and or RE was used for intervention; (c) a comparable control group applied with other treatments, including health education and sham training; (d) outcomes including skeletal muscle mass related parameters (body mass index, BMI; fat-free mass index, FFM; and cross-sectional area, CSA), strength-related parameters (isometric, isotonic, and isokinetic strength), endurance exercise capacity (6-min walking distance, 6MWD), and peak exercise capacity (peak oxygen consumption, VO_2peak_); and (e) randomized controlled study published in English. The exclusion criteria were as follows: (a) the immediate response to a single exercise test or exercise session was studied; (b) the follow-up effects of previous exercise program were studied; (c) traditional Chinese exercise and yoga were used for interventions; (d) animal trials, observational trials, expert opinions, literature reviews, comments, and letters were involved; (e) regular exercise programs were utilized in control groups (e.g., breath training, ≥twice a week); and (f) data could not be extracted.

Electronic searches of PubMed, Embase, EBSCO, Web of Science, and PEDro database were conducted from inception to April 25, 2020 using Medical Subject Headings (MeSH) terms and free-text keywords. In addition to the PEDro database, the following search terms were used: (COPD OR chronic obstructive pulmonary disease OR chronic obstructive lung disease OR chronic obstructive airway disease) AND (exercise OR exercise training OR rehabilitation OR pulmonary rehabilitation OR aerobic exercise OR endurance exercise OR resistance exercise OR strength training OR combined exercise) AND (muscle OR skeletal muscle). Search filters were applied, including article type (randomized controlled trials), species (humans), and language (English). In the PEDro database, the search terms were as follows: topic (chronic respiratory disease), method (clinical trial), therapy (fitness training), and abstract and title (COPD). Searches were supplemented by reviewing the reference lists of the included studies, previous review, meta-analysis, and guidelines.

To determine the eligibility of identified studies, two investigators independently conducted the process of study selection. Cohen's kappa was used to quantify the interrater agreement. Discrepancies of opinion between authors about study eligibility were resolved through discussions with a third investigator.

### Data Analysis

Two investigators independently extracted data on study design, sample characteristics, intervention programs, and effects of exercise from included studies. Discrepancies were resolved through discussions with a third investigator. The studies were described in terms of study design (sample size, and PEDro score), sample characteristics (age, sex, FEV1%pred for forced expiratory volume in 1 s, and BMI), intervention programs (site, exercise modality, intensity, frequency, and duration), effects of exercise (outcome measures and change data), and adherence to the program. For trials with more than one exercise intervention group, the effects of each exercise intervention were evaluated. For trials with more than one outcome measures, the data of each outcome measures was included and analyzed. For trials with multiple time points, only the pre-intervention and post-intervention outcomes were extracted.

Predetermined primary outcomes included skeletal muscle mass (BMI, FFM, and CSA), strength (isometric, isotonic, and isokinetic strength), endurance exercise capacity (6MWD), and peak exercise capacity (VO_2peak_). Secondary outcomes were attrition rate and severe adverse events. The change in mean and SD were calculated for each outcome and used to estimate the effects of the exercise. Summary measures for continuous outcomes were mean difference (MD) or standard mean difference (SMD) with 95% CI, and odds ratio (OR) with 95% CI for the attrition rate.

Review Manager (version 5.3) provided by Cochrane was used for meta-analysis. Random-effects model was used for analyzing. The *I*^2^ statistic, representing the percentage of variation across studies due to heterogeneity, was used to assess heterogeneity between studies. Planned subgroup analyses were conducted in terms of exercise modalities (EE, RE, and CE) and outcome measures (isometric, isotonic, and isokinetic strength test). Sensitivity analyses were performed to check the heterogeneity source based on the intervention program and characteristics of the participants when subgroup analysis could not determine the source of substantial heterogeneity. Visual inspection of funnel plots and Egger's test were undertaken in Stata (version 15) to assess publication bias. Trim and fill method was used when there was a publication bias. The methodological quality of randomized controlled trial (RCTs) was assessed using the physiotherapy evidence database (PEDro) scale. When available, the PEDro rating and score were obtained from the PEDro database. Otherwise, two investigators independently rated and scored the publications; discrepancies were resolved through discussions with a third investigator. The PEDro scale includes 11 items with 10 scores, and a higher score means better quality (19). It should be noted that the eligibility criteria item does not contribute to the total score. PEDro scale 9–10 was considered high quality, 6–8 was generally high quality, 4–5 was moderate quality, and <4 was low quality. The quality of evidence was assessed according to the Grading of Recommendations Assessment, Development, and Evaluation (GRADE) recommendations (limitation of study design, inconsistency, indirectness, imprecision, and publication bias) (20).

## Results

**Figure 1:**
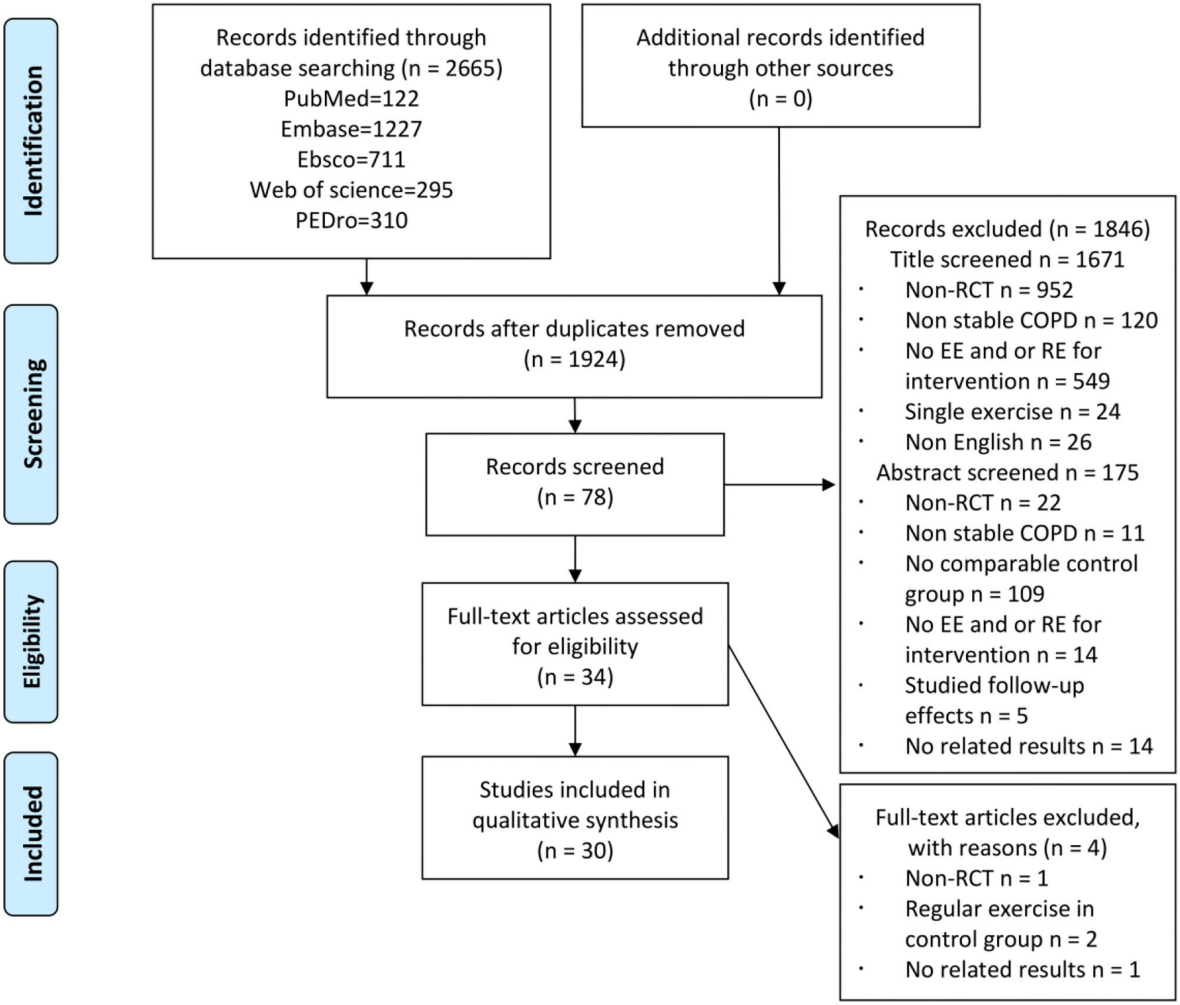
Study flow diagram. COPD, chronic obstructive pulmonary disease; EE, endurance exercise; RCT, randomized controlled trial; RE, resistance exercise.

A total of 2,665 records were identified, and 30 RCTs were included in the quantitative analysis (Figure 1). A strong agreement was observed with respect to the interrater reliability of study selection (kappa = 0.89, *P* < 0.001). The PEDro scale of all included studies is 5.7 ± 1.4 (Supplementary Table S1), and the characteristics of participants of each included study are reported in Table 1. A total of 1,317 participants with stable COPD (age range from 46 to 79.8 years) were included, and 675 (51%) participants accepted exercise intervention. According to the criteria of Global strategy for the diagnosis, management, and prevention of COPD (GOLD), majority of the participants showed moderate to severe airflow restriction (30% ≤ FEV1%pred ≤ 80%), and four studies did not provide the baseline data of FEV1%pred (27, 30, 38, 41). Most participants were normal to overweight (BMI: 18.5–29.9 kg/cm^2^), while five studies did not provide this data (27, 38, 39, 42, 49). In addition, exercise intervention programs of all the included studies are presented in Table 2. Most trials were conducted in a hospital, at home, or at both the places, while six studies did not report a place (31, 35, 38, 40, 45, 50). Most studies applied exercise program duration ranges from 6 to 12 weeks, while some studies applied 14 weeks (22), 16 weeks (47), and 24 weeks (38). EE was mainly performed in the form of treadmill, cycling, or walking with a moderate to vigorous exercise intensity (Borg 4–6, even exhaustion, despite indexes used to assess were various) for two to three sessions per week. RE was mainly performed on weight machines, free weights, and elastic bands through the movements of the upper and lower limbs. One study performed RE only through the upper limbs (35) and three studies conducted RE only through the lower limbs (31, 33, 34). Exercise intensity of RE ranged from 50 to 85% 1-repetition maximum (1RM) or Borg 4–6, and exercise frequency was two to three sessions per week. The performance of CE was consistent with EE and RE. The exercise intensity of EE was Borg 4–6, while the exercise intensity of RE was often unclear. The characteristics of muscle strength testing relative to the variety of muscle strength testing methods and programs are summarized in Table 3.

**Table 1:** Characteristics of included studies.

**Author, Country**	**I/C sample size/Male%**	**Age**	**FEV1%pred**	**BMI**	**Outcome**			Attrition number^*^	**PEDro**
					**Mass**	**Strength**	**Exercise capacity**		
***Endurance exercise***									
Alcazar et al. (21), Spain	14(79%)/ 15(87%)	77.7 ± 7.9/ 79.8 ± 6.4	47.4 ± 18.1/ 58.7 ± 15.2	28.8 ± 3/ 32.5 ± 5.9	Mid-thigh CSA	Leg press	6MWD VO_2peak_	5/1^*^	4
Barakat et al. (22), France	40(85%)/ 40(83%)	63.7 ± 11.9/ 65.9 ± 10.3	41.9 ± 2.6/ 43.33 ± 3.6	24.2 ± 6.4/ 25.6 ± 4.3	/	/	6MWD	5/4^*^	6
Borghi-silva et al. (23), USA	20(65%)/ 14(86%)	67 ± 10/ 67 ± 10	33 ± 9/ 35 ± 11	25 ± 4/ 24 ± 5	/	/	6MWD VO_2peak_	0/6	5
Borghi-silva et al. (24), USA	10(70%)/ 10(50%)	67 ± 7/ 66 ± 10	32 ± 11/ 35 ± 12	23.4 ± 4.4/ 27.2 ± 4.7	/	/	6MWD VO_2peak_	7/5	5
de Souto Araujo et al. (25), Brazil	13(62%)/ 11(73%)	56.9 ± 7.9/ 71.1 ± 10.1	39.2 ± 11.4/ 45.1 ± 12.6	30 ± 10.1/ 24.4 ± 6.7	/	/	6MWD	1/3	4
Gallo-Silva et al. (26), Brazil	10/9	66.3 ± 6.5/ 66.5 ± 9.5	47.9 ± 20.5/ 47.8 ± 26.2	23.2 ± 2.6/ 25.7 ± 6.1	/	/	6MWD	2/3	6
Mehri et al. (27), Iran	20(55%)/ 18(39%)	52.1 ± 10.7/ 52.17 ± 11.6	/	/	/	/	VO_2peak_	0/0	5
Petersen et al. (28), Denmark	9(22%)/ 10(40%)	67 ± 3/ 66 ± 3	33 ± 5/ 30 ± 4	23 ± 2/ 26 ± 2	/	/	VO_2_ max	0/4	5
Pradella et al. (29), Brazil	29(79%)/ 15(87%)	62.4 ± 10.7/ 65.3 ± 8	43.9 ± 16.2/ 54 ± 22.2	25.2 ± 5/ 26.7 ± 5.3	/	/	6MWD	3/3	5
Wiyono et al. (30), Indonesia	27(93%)/ 29(96%)	64.3 ± 6.3/ 67.2 ± 4.5	/	19.7 ± 8.5/ 20.2 ± 6.4	/	/	6MWD VO_2_ max	3/1	5
***Resistance exercise***									
Casaburi et al. (31), USA	12 (100%)/ 12(100%)	68.9 ± 9.8/ 67.7 ± 8.7	35.9 ± 9.2/ 38.6 ± 12.1	27.57/ 26.31	Total lean mass	Leg press	VO_2peak_	1/1	5
Clark et al. (32), Scotland	26(58%)/ 17(59%)	51 ± 10/ 46 ± 11	76 ± 23/ 79 ± 23	26 ± 4/ 26 ± 4	BMI	Quadriceps	VO_2_ max		5
Chen et al. (33), China	25(88%)/ 22(68%)	69.04 ± 8.07/ 64.95 ± 11.59	54.49 ± 23.62/ 54.93 ± 25.58	23.86 ± 3.9/ 24.15 ± 3.93	/	Quadriceps	6MWD	4/4	6
Hoff et al. (34), USA	6(67%)/ 6(67%)	62.8 ± 1.4/ 60.6 ± 3	32.9 ± 3.3/ 39.5 ± 6.4	26.27/ 26.65	BMI	Leg press	VO_2peak_	0/0	6
Janaudis-Ferreira et al. (35), Canada	17(53%)/ 19(37%)	67 ± 11/ 67 ± 11	37.8 ± 16.2/ 32.5 ± 14.1	27.9 ± 7.9/ 25.7 ± 8.2	/	Biceps Triceps Anterior Middle deltoids	/	4/1^*^	9
Nyberg et al. (36), Sweden	22(55%)/ 22(50%)	69 ± 5/ 68 ± 6	59 ± 11/ 55 ± 15	26 ± 4/ 25 ± 5	/	Shoulder flexion Knee extension	6MWD	2/2^*^	8
O'shea et al. (37), Australia	27/27	66.9 ± 7/ 68.4 ± 9.9	49 ± 25/ 52 ± 22	25.5 ± 5.1/ 27.8 ± 7.9	/	Knee extension Hip abduction Shoulder horizontal flexion Shoulder flexion	6MWD	7/3^*^	7
Thabitha et al. (38), India	30	/	/	/	/	/	6MWD VO_2peak_	/	4
Simpson et al. (39), Canada	14(35%)/ 14(71%)	73 ± 4.8/ 70 ± 5.7	39.5 ± 18.96/ 39.2 ± 21.39	/	/	Elbow flexion Quadriceps Leg press	6MWD VO_2_ max	3/3	6
Zambom-Ferraresi et al. (40), Spain	14(100%)/ 8(100%)	68 ± 7/ 69 ± 5	48 ± 12/ 39.7 ± 5	28.5 ± 3.9/ 25.7 ± 4.6	/	Leg press Chest press	6MWD VO_2peak_	1/1	7
***Combined exercise***									
Cameron-Tucker et al. (41), Australia	43(53%)/ 41(54%)	64.5 ± 9.3/ 67.1 ± 9.41	/	28.4 ± 7.63/ 29.7 ± 6.5	/	/	6MWD	5/10^*^	6
Emery et al. (42), USA	30(50%)/ 24(42%)	65.4 ± 6.4/ 67.4 ± 5.9	43 ± 18/ 43 ± 18	/	/	/	VO_2_ max	4/2^*^	6
Lahham et al. (43), Australia	29(59%)/ 29(59%)	68 ± 9/ 67 ± 10	90 ± 8/ 92 ± 7	28 ± 4.5/ 28 ± 4.3	/	/	6MWD	3/4^*^	8
Mendes et al. (44), Brazil	23(83%)/ 29(66%)	71.3 ± 6.7/ 70.8 ± 8.7	51.5 ± 23.9/ 41.4 ± 18.4	23.5 ± 4.2/ 24.6 ± 6.3	/	/	6MWD	23/0	4
Nakamura et al. (45), Japan	10/ 10	69 ± 8.7/ 69.9 ± 7.1	53.2 ± 15.1/ 48.2 ± 20.1	21.9 ± 3.5/ 21.6 ± 3	/	HGF	6MWD VO_2peak_	/	5
Tsai et al. (46), Australia	19(63%)/ 17(35%)	78 ± 3/ 75 ± 9	60 ± 23/ 68 ± 19	28 ± 4/ 28 ± 5	/	/	6MWD	1/0	8
van Wetering et al. (47), Netherlands	102(71%)/ 97(71%)	65.9 ± 8.8 / 67.2 ± 8.9	58 ± 17/ 60 ± 15	26.1 ± 4.4/ 27.3 ± 4.7	BMI FFMI	HGF Quadriceps	6MWD	15/9^*^	7
Wadell et al. (48), Canada	17(53%)/ 24(54%)	68 ± 6/ 66 ± 7	48 ± 12/ 48 ± 19	26.7 ± 4.9/ 28.9 ± 4.3	/	Knee extension	6MWD	3/ 4^*^	6
Wadell et al. (49), Sweden	15(33%)/ 13(54%)	65 ± 7/ 63 ± 7	53 ± 12/ 49 ± 12	/	/	/	VO_2peak_	1/ 1^*^	6
Weiner et al. (50), Israel	18/ 5	63.2 ± 2.3/ 60.1 ± 2.8	35 ± 2.2/ 36 ± 1.9	23.84/ 24.84	/	/	6MWD	1/1^*^	5
Zambom-Ferraresi et al. (40), Spain	14(100%)/ 8(100%)	68 ± 7/ 69 ± 5	44.3 ± 11.9/ 39.7 ± 5	29.3 ± 6.4/ 25.7 ± 4.6	/	Leg press Chest press	6MWD VO_2peak_	2/1	7

*6MWD, 6-min walking distance; HGF, Handgrip force; I/C, Intervention group/Control group; RM, Repetition maximum; VO_2_, Oxygen uptake*.

*/Not accessible;*

* *Attrition number is included in the sample size*.

**Table 2:** Characteristics of intervention protocols.

**Author, Country**	**Setting**	**Intervention contents**	**Intervention intensity**	**Intervention duration/frequency**	**Control group**
***Endurance exercise***					
Alcazar et al. (21), Spain	Outpatient	First 3 weeks: HIIT (5 sets of 90 s at light intensity plus 30 s at heavy intensity) + power training (2–3 sets of 8–12 reps) Week 4–12: HIIT (10stes) + 3sets of 8reps with the optimal load	First 3 weeks: HIIT (heavy-80%*W*_peak_, light-40% *W*_peak_) + power training (50–60% 1RM) Week 4–12: HIIT (augmented) + power training (optimal load)	2 sessions/week, 12 weeks	Usual care
Barakat et al. (22), France	Outpatient	30 min cycling + 30 min aerobic activity (5 min warm-up, 10 min aerobic activity, 15 min cool-down)	Cycle: 80%VO_2_ max	3 sessions/week, 14 weeks	Routine outpatient attendance
Borghi-Silva et al. (23), USA	Outpatient	30 min stretching + treadmill ambulation	70% of the maximal speed	3 sessions/week, 6 weeks	Usual care
Borghi-Silva et al. (24), USA	Outpatient	5 min warm-up + 30 min treadmill	70% of the peak speed/Borg 4	3 sessions/week, 12 weeks	Respiratory therapy, 1session/week
de Sauto Araujo et al. (25), Brazil	Outpatient	15 min callisthenic activities + 30 min unsupported upper limb exercise using weights + 30 min bicycle + 15 min cool-down	Upper: 50% of the maximum load; Lower: Borg 5	3 sessions/week, 8 weeks	No exercise
Gallo-Silva et al. (26), Brazil	Laboratory	60 min water aerobic interval exercise (10 min warm-up, 20–40min aerobic exercise, 10 min cool-down)	Borg 4–6	3 sessions/week, 8 weeks	Usual care
Mehri et al. (27), Iran	Outpatient	Treadmill exercise training with gradually increased speed	Exhaustion	2 sessions/week, 8 weeks	No exercise
Petersen et al. (28), Denmark	Outpatient	Walking with 85% maximal speed + progressive ergometer cycling	Exhaustion	2 sessions/week, 7 weeks	Usual daily activities
Pradella et al. (29), Brazil	Home	40 min walking + 15 min stair exercise + arm exercise with 1 kg load (3 sets of 30 movements)	Walking: 60–70% HR_max_	3 sessions/week, 8 weeks	No exercise
Wiyono et al. (30), Indonesia	Outpatient	5 min cycling, and gradually increased for 5 min/week	/	3 sessions/week, 6 weeks	Routine outpatient attendance
***Resistance exercise***					
Casaburi et al. (31), USA	/	First 4 weeks: 3 sets of 12 reps; Week 5–10: 4 sets of 8–10 reps (seated leg press, seated leg curl, seated leg extension, standing calf raise, seated ankle dorsiflexion)	First 4 weeks: 60% 1RM Week 5–10: 80% 1RM	3 sessions/week, 10 weeks	No exercise
Clark et al. (32), Scotland	Outpatient	3 sets of 10 reps weight exercises (bench press/triceps, body squat/quadriceps, squat calf/medial and lateral gastrocnemiius soleus, latissimus/latissimus dorsi/arm curls/biceps, leg press/quadriceps hamstrings gluteals, knee flexion/quadriceps, hamstrings)	70% maximal load	2 sessions/week, 12 weeks	Usual daily activities
Chen et al. (33), China	Home	20–30min, 8–12 reps Thera-band exercise (straight-leg lifting, prone hip extension, thigh abduction, posterior muscle group exercises, anterior muscle group exercises, and standing calf raise)	Borg 5	3 sessions/week, 12 weeks	No exercise
Hoff et al. (34), USA	Laboratory	4 sets of 5 reps concentric contraction of quadriceps	85–90% 1RM	3 sessions/week, 8 weeks	Normal daily living
Janaudis-Ferreira et al. (35), Canada	/	10–12RM using free weights and a multistation gym (biceps brachii, triceps brachii, pectoralis major and minor, latissimus dorsi, deltoids, rhomoboids)	10–12RM	3 sessions/week, 6 weeks	Upper limb flexibility and stretching exercises
Nyberg et al. (36), Sweden	Outpatient	40 min, 2 sets of 25 reps Thera-band exercise (Latissimus row/chest press/leg extension/shoulder flexion/leg curl/elbow flexion/heel-raise/step up)	Borg 4	3 sessions/week, 8 weeks	No exercise
O'shea et al. (37), Australia	1 hospital + 2 home	3 sets of 8–12 reps Thera-band exercise (hip abduction in standing, simulated lifting, SST, seated row, lunges, chest press)	12RM and gradually increased	3 sessions/week, 12 weeks	No exercise
Thabitha et al. (38), India	/	15–30min, 1–3 sets of 10 reps using multi exerciser (chest pull-lattismus dorsi, butterfly-pectoralis major muscle, neck press-triceps brachii and deltoid, leg flexion-biceps femoris and gastronemious, leg extension)	10RM and increased by 10%	2 sessions/day, 3 days/week, 24 weeks	No exercise
Simpson et al. (39), Canada	Outpatient	3 sets of 10 reps single limb weight lifting exercise (arm curl/leg extension/leg press)	50–85% 1RM	3 sessions/week, 8 weeks	No exercise
Zambom-Ferraresi et al. (40), Spain	/	90 min, 3–4 sets of 6–12 reps (chest press, seated row, shoulder press, leg press, knee extension and flexion)	50–70% 1RM	2 sessions/week, 12 weeks	Habitual physical activity
***Combined exercise***					
Cameron-Tucker et al. (41), Australia	Outpatient	1 h combine exercises, individualized for each participant	RPE 3–5	1 sessions/week, 6 weeks	No exercise
Emery et al. (42), USA	Outpatient	First 5 weeks: 45 min combine exercises on Nautilus equipment; Week 6–10: 60–90 min	/	First 5 weeks: every-day; Week 6–10: 3 sessions/week	No exercise
Lahham et al. (43), Australia	Home	Aerobic: 80% of walking speed from 6MWD + 30 min whole-body exercise; Resistance: using equipment available at home (stairs and sealed water bottles)	/	5 sessions/week, 8 weeks	No exercise
Mendes et al. (44), Brazil	Outpatient	Aerobic: 30 min treadmill walking; Resistance: 10 reps (hand weight, elbow flexion, elbow abduction, shoulder abduction, shoulder flexion, hip flexion, knee extension)	Aerobic: 60–80% HR_max_ Resistance: 50% 1RM with an increase of 0.5 kg every 2 weeks	3 sessions/week, 12 weeks	No exercise
Nakamura et al. (45), Japan	/	Aerobic: 20 min walking; Resistance: 30 min, 3 sets of 10 reps using self-weight or elastic bands (push-ups, leg squats, sit-ups, back extension)	Aerobic: Borg 3–5	12 weeks	No exercise
Tsai et al. (46), Australia	Home	Aerobic: 15–20 min cycling + 15–20 min walking Resistance: 3 sets of 10 reps SST and squats exercise	Cycle: 60–80% *W*_peak_ Walk: 80% of best 6MWD or Aerobic: Borg 3–4	3 sessions/week, 8 weeks	Usual care
van Wetering et al. (47), Netherlands	Community	Aerobic: 30 min cycling/walking Resistance: 4 specific exercises for upper and lower limbs	/	2 sessions/week, 16 weeks	Usual care
Wadell et al. (48), Canada	Outpatient	2.5 h combine exercise	Moderate intensity	3 sessions/week, 8 weeks	Usual care
Wadell et al. (49), Sweden	Outpatient	45 min, (4 min aerobic, 3 min leg resistance, 4 min aerobic, 3 min arm resistance, 4 min aerobic, 3 min torso resistance)	80–100%HR peak or Borg 5 or RPE 15	3 sessions/week, 12 weeks	No exercise
Weiner et al. (50), Israel	/	Aerobic: 30 min cycling; Resistance: 15 min rowing with low resistance +15 min resistance exercises for limbs and abdominal muscles	Aerobic: 50% *W*_max_	3 sessions/week, 6 weeks	Sham training
Zambom-Ferraresi et al. (40), Spain	/	Aerobic: 20–35 min cycle Resistance: 90 min, 3–4 sets of 6–12 reps (chest press, seated row, shoulder press, leg press, knee extension and flexion)	Aerobic: 40–85% *W*_max_ Resistance: 50–70% 1RM	2 sessions/week for each exercise types, 12 weeks	Habitual physical activity

*6MWD, 6-min walking distance; HIIT, high intensity interval training; HGF, Handgrip force; HR, Heart rate; reps, repetitions; RM, Repetition maximum; SST, sit to stand; W_max_, Maximal work rate; VO_2_, Oxygen uptake*.

*/Not accessible*.

**Table 3:** Characteristics of skeletal muscle strength tests.

**Type**	**Author, Country**	**Outcomes**	**Methods**	**Apparatus**	**Site**
Isometric test	Alcazar et al. (21), Spain	Leg press (N)	Evaluate two legs performance, test for at least 4s	Force plate	Lower limb
	Chen et al. (33), China	Quadriceps (Nm)	Evaluate the maximal strength of dominant leg	Computerized dynamometer	Lower limb
	Janaudis-Ferreira et al. (35), Canada	Biceps (kg) Triceps (kg) Anterior (kg) Middle deltoids (kg)	Evaluate the dominant side by Micro FET2, the average of the highest 3 measures were used for analysis	Hand-held dynamometer	Upper limb
	Nakamura et al. (45), Japan	HGF (kg)	Evaluate the dominant side	Hand-grip dynamometer	Upper limb
	Wadell et al. (48), Canada	Knee extension (kg)		Fixed dynamometer	Lower limb
	van Wetering et al. (47), Netherlands	HGF (kg) Quadriceps (Nm)		Unknown device	Upper limb Lower limb
Isotonic test	Casaburi et al. (31), USA	Leg press (kg)	Evaluate two legs performance by 1RM test	Pneumatic device	Lower limb
	Clark et al. (32), Scotland	Quadriceps (kg)	1RM test	Multigym	
	Hoff et al. (34), USA	Leg press (kg)	1RM test	Force platform	
	O'shea et al. (37), Australia	Knee extension (kg) Hip abduction (kg) Shoulder horizontal flexion (kg) Shoulder flexion (kg)	Averaged across right and left limbs were used for analysis	Hand-held dynamometry	Lower limb Lower limb Upper limb Upper limb
	Simpson et al. (39), Canada	Elbow flexion (kg) Quadriceps (kg) Leg press (kg)	Unilateral 1RM test	Unknown device	Upper limb Lower limb
	Zambom-Ferraresi et al. (40), Spain	Leg press (kg) Chest press (kg)	1RM test	Force plate	Lower limb Upper limb
Isokinetic test	Chen et al. (33), China	Quadriceps (Nm)	Evaluate the maximal strength of dominant leg	Computerized dynamometer	Lower limb
	Nyberg et al. (36), Sweden	Shoulder flexion (Nm) Knee extension (Nm)	The highest of 5 maximal contractions was used for analysis	Computerized dynamometer	Upper limb Lower limb

*Kg, Kilogram; HGF, Handgrip force; N, Newton; RM, Repetition maximum*.

**Figure 2:**
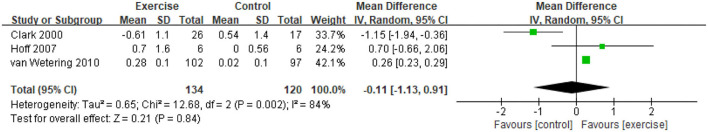
Pooled effect of exercise on BMI in patients with COPD. BMI, body mass index (kg/m^2^); CI, confidence interval; COPD, chronic obstructive pulmonary disease; SD, standard deviation.

**Figure 3:**
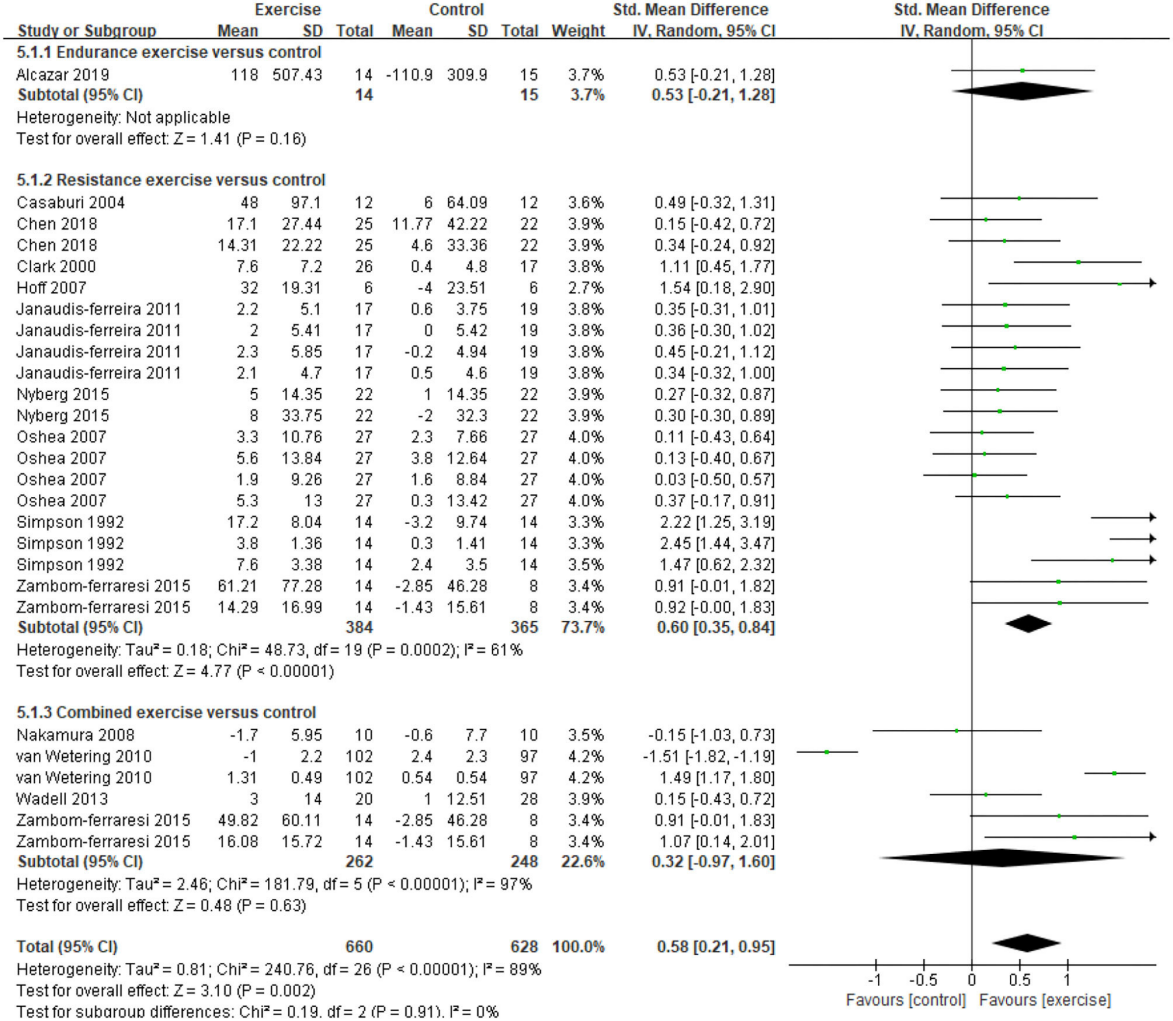
Effects of three types of exercise on skeletal muscle strength in patients with COPD. CI, confidence interval; COPD, chronic obstructive pulmonary disease; SD, standard deviation.

**Figure 4:**
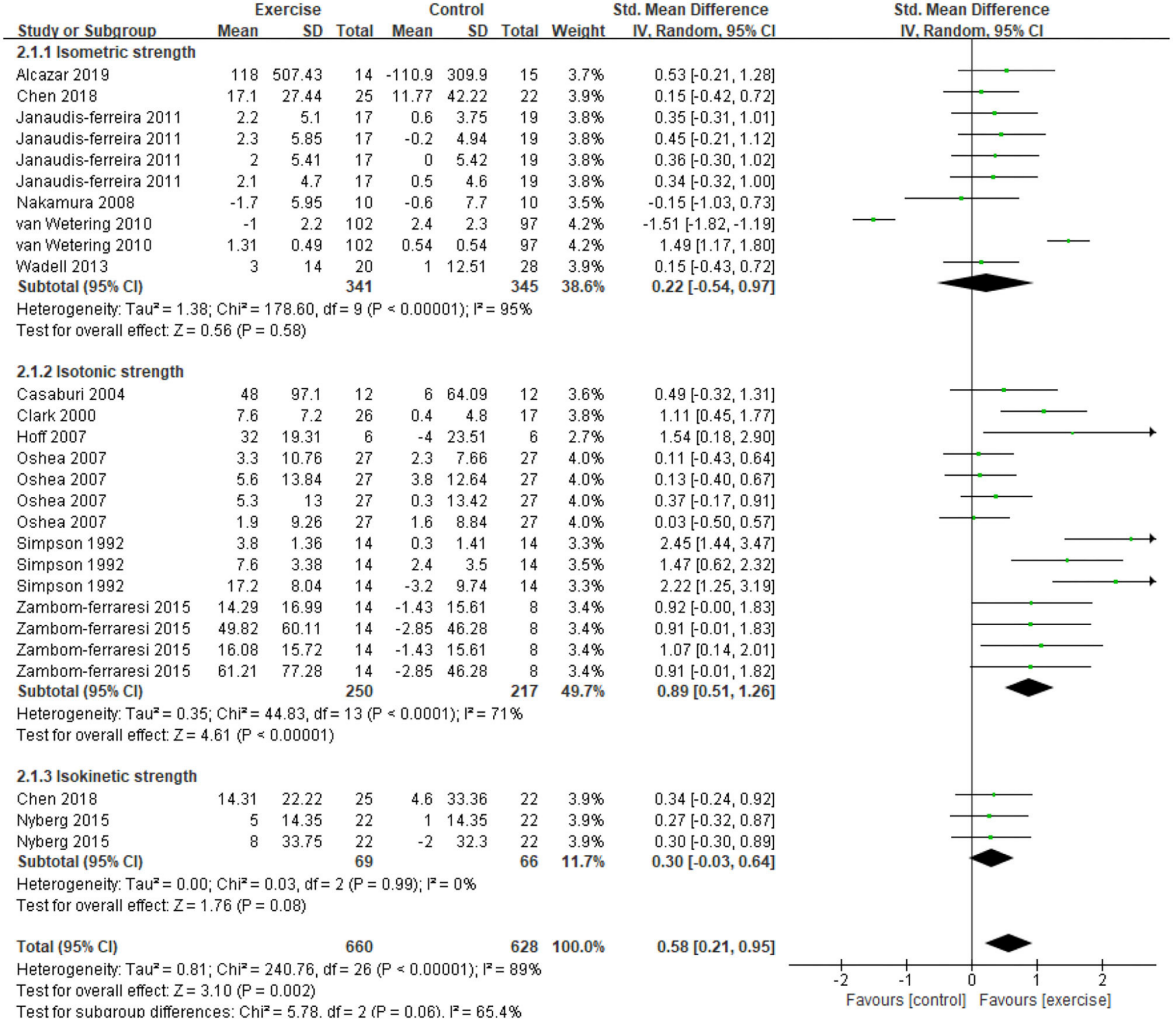
Effects of exercise on skeletal muscle strength evaluated by three types of measurements in patients with COPD. CI, confidence interval; COPD, chronic obstructive pulmonary disease; SD, standard deviation.

Five studies (21, 31, 32, 34, 47) provided data on skeletal muscle mass, assessed by mid-thigh CSA, BMI, FFMI, and total lean mass. In the meta-analysis, the estimated results showed that exercise intervention did not have a significant effect on changes in BMI (MD = −0.11, 95% CI: 1.13–0.91, *I*^2^ = 84%, Figure 2). Considering the high heterogeneity detected, we excluded studies with PEDro <6, and found a significant improvement in BMI (MD = 0.26, 95% CI 0.23–0.29, *I*^2^ = 0%). In addition, a CE program significantly improved FFMI (*P* = 0.01) (47), an EE program significantly improved the mid-thigh CSA (+4.5%, *P* < 0.05) of elderly patients with COPD (age: 77.7 ± 7.9 years old) (21), an RE program only found an increasing trend in the total lean mass (31). A total of 13 studies (21, 31–37, 39, 40, 45, 47, 48) with 27 data on skeletal muscle strength were provided, demonstrating a significant improvement after exercise intervention (SMD = 0.58, 95% CI 0.21–0.95, *I*^2^ = 89%). Considering the high heterogeneity detected, we first excluded studies with PEDro <6, and found a consistent result with high heterogeneity (SMD = 0.62, 95% CI 0.19–1.05, *I*^2^ = 91%). Then, we only pooled data in kilograms unit, and found a consistent result (MD = 0.78, 95% CI 0.64–0.92, *I*^2^ = 0%) besides the isometric strength test. Finally, subgroup analysis for different exercise modalities (Figure 3), muscle strength measures (Figure 4), and upper or lower limbs muscle strength found that RE provided significant benefits (SMD = 0.6, 95% CI 0.35–0.84, *I*^2^ = 61%), isometric strength significantly improved (SMD = 0.89, 95% CI 0.51–1.26, *I*^2^ =71%), and both upper and lower limbs muscle strength significantly improved (SMD = 0.78, 95% CI 0.4–1.17, *I*^2^ = 79%; SMD = 0.67, 95% CI 0.12–1.22, *I*^2^ = 91%).

**Figure 5:**
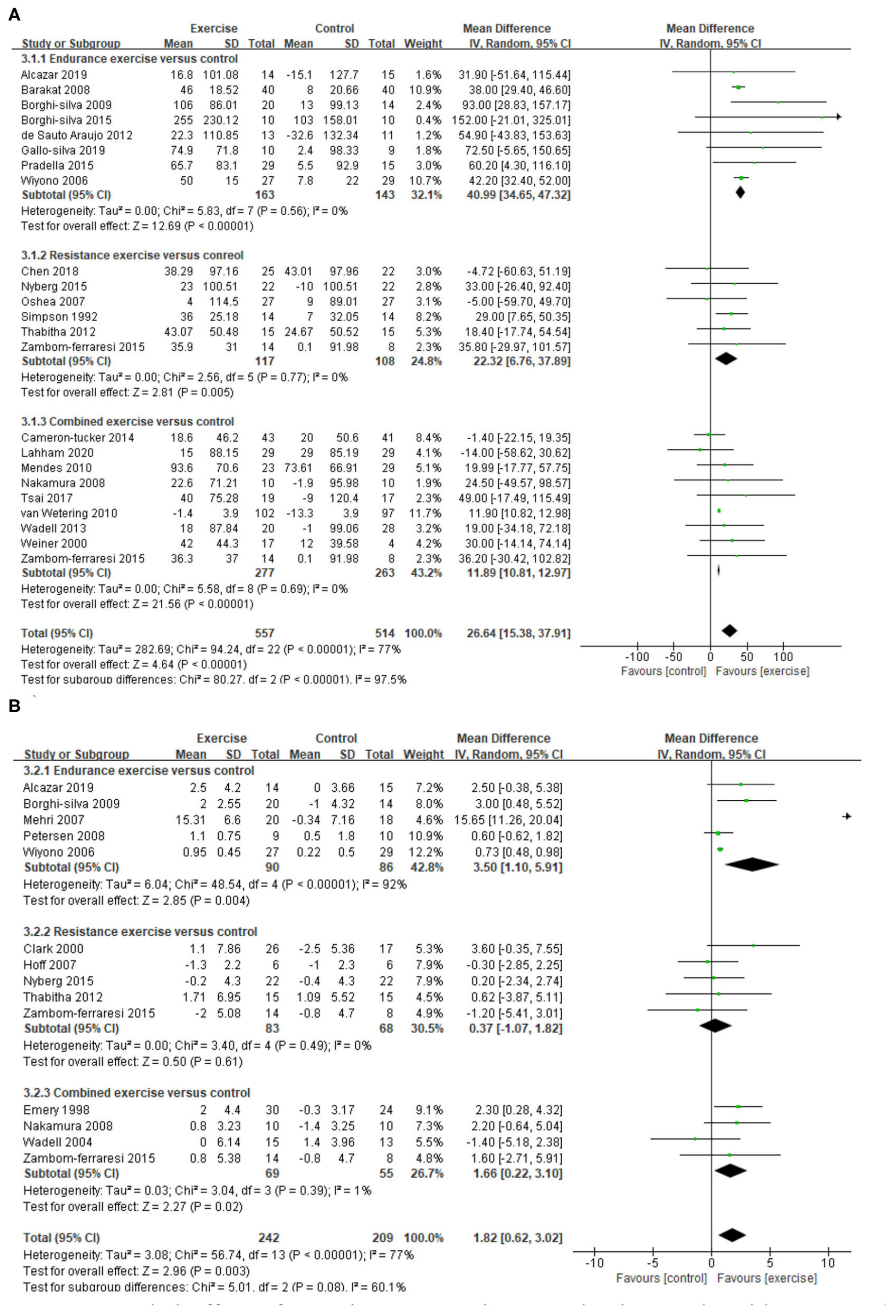
Pooled effect of exercise on exercise capacity in people with COPD. **(A)** 6MWD, **(B)** VO_2peak_. 6MWD, 6-min walking distance (m); CI, confidence interval; COPD, chronic obstructive pulmonary disease; SD, standard deviation; VO_2peak_, peak oxygen consumption (ml/kg/min).

A total of 22 studies (21–26, 29, 30, 33, 36–41, 43–48, 50) provided data on endurance exercise capacity, demonstrating a significant improvement in 6MWD after exercise intervention (MD = 26.64, 95% CI 15.38–37.91, *I*^2^ = 77%). Subgroup analysis for different exercise modalities showed a consistent result, namely that all EE, RE, and CE can improve 6MWD significantly (EE: MD = 40.99, 95% CI 34.65–47.32, *I*^2^ = 0%; RE: MD = 22.32, 95% CI 6.76–37.89, *I*^2^ = 0%; CE: MD=11.89, 95% CI 10.81–12.97, *I*^2^ = 0%, Figure 5). A total of 13 studies (21, 23, 27, 28, 30, 32, 34, 36, 38, 40, 42, 45, 49) provided data on the peak exercise capacity, demonstrating a significant improvement in VO_2peak_ after exercise intervention (MD = 1.82, 95% CI 0.62–3.02, *I*^2^ =77%). Subgroup analysis for different exercise modalities showed that EE and CE can improve VO_2peak_ significantly (EE: MD = 3.5, 95% CI 1.1–5.91, *I*^2^ =92%; CE: MD = 1.66, 95% CI 0.22–3.1, *I*^2^ =1%, Figure 5). Considering that the methodological quality of included studies in EE was relatively low (PEDro <6), the results need to be carefully considered.

There was no difference of attrition number between exercise and control group (OR = 1.12, 95% CI 0.75–1.67, *I*^2^ = 15%, Supplementary Figure S1). The reasons for attrition in the exercise and control groups were similar (Supplementary Table S2).

Funnel plots are presented in Supplementary Figure S2. The results of Egger's test showed a significant publication bias in the results of skeletal muscle strength and 6MWD (*P* = 0.031 and *P* = 0.018, respectively). Then, the trim and fill method was used to adjust the impact of publication bias, and the results showed 0 missing studies for skeletal muscle strength results, and five missing studies for 6MWD results were merged to diminish the publication bias (Supplementary Figure S3). The certainty of the evidence for endurance and peak exercise capacity was deemed moderate, for skeletal muscle strength was deemed low, and for BMI was deemed very low (Table 4).

**Table 4:** Grading of recommendations assessment, development, and evaluation summary of findings.

**Certainty Assessment**								**No. of Patients**		**Effect**		**Certainty**
**Outcomes**	**No. of Studies**	**Study design**	**Risk of bias**	**Inconsistency**	**Indirectness**	**Imprecision**	**Other considerations**	**EG**	**CG**	**Relative (95% CI)**	**Absolute (95% CI)**	
BMI	3	Randomized trials	Serious^a^	Serious^b^	Not serious	Serious^c^	None	134	120	–	MD 0.11 lower (1.13 lower to 0.91 higher)	⊕◯◯◯ Very low
Skeletal muscle strength	13	Randomized trials	Serious^a^	Serious^b^	Not serious	Not serious	None	660	628	–	SMD 3.48 higher (1.81 to 5.15 higher)	⊕⊕◯◯ Low
6MWD	22	Randomized trials	Serious^a^	Not serious	Not serious	Not serious	None	557	514	–	MD 12.76 higher (11.69 to 13.82 higher)	⊕⊕⊕◯ Moderate
VO_2peak_	13	Randomized trials	Serious^a^	Not serious	Not serious	Not serious	None	242	209	–	MD 1.82 higher (0.62 to 3.02 higher)	⊕⊕⊕◯ Moderate

a *Most of the studies are without allocation concealment, subject blinded and intention-to-treatment analysis*.

b *There was a substantial heterogeneity among the three studies according to the heterogeneity test*.

c *Only three studies were included in the analysis, and the sample size was relatively low*.

## Discussion

This systematic review and meta-analysis confirmed that regular exercise intervention for more than 6 weeks can effectively improve peripheral skeletal muscle strength and exercise capacity of patients with stable COPD. Furthermore, the greatest improvement in peripheral skeletal muscle strength appears in RE, the greatest improvement in endurance exercise capacity (6MWD: 40.99 m) appears in EE, and both EE and CE can significantly improve the peak exercise capacity.

In a previous study, skeletal muscle wasting could occur in the early COPD stages (51), and different exercise modalities could effectively improve lower limb muscle mass in COPD (14). However, in this study, exercise significantly improved the BMI of patients with COPD after excluding studies with PEDro <6. Through the analysis of literature characteristics, we proposed that exercise improved the BMI of patients with COPD unrelated to exercise modalities, but it was more affected by age and FEV1%pred. That is, the younger the age and better FEV1%pred, the lower the potential for improvement by exercise intervention. A recent meta-analysis of clinical trials has found a negative correlation between the BMI and decline of FEV1 in patients with COPD (52). Age, severity of COPD, and dyspnea degree are closely and clinically related to the loss of skeletal muscle mass and the decline of muscle function in patients with COPD (51). The results from the above-mentioned cross-sectional trials supported the speculation, but the factors that modulated the effects of exercise in COPD skeletal muscle mass still need to be explored due to the small data size in this study. Furthermore, BMI is affected by adipose and connective tissues in the body and may inadequately reflect muscle mass changes. Previous studies have found that RE can significantly improve lower limb lean muscle mass, increase the CSA of the rectus femoris and quadriceps, and decrease the density of muscle fiber (which indicate increased muscle mass per unit area) in COPD (53, 54), but have no effects on the proportion of muscle fiber type and the CSA of different muscle fiber types (an increasing trend only be found in type IIx fibers) (54). Another trial compared the effects of EE and RE on quadriceps muscle morphology and found no significant change in proportion and CSA of type I fibers, intermediate fibers, type IIx fibers, and capillarization (expressed as capillary-to-fiber ratio capillary density) after both exercise modalities, while the proportion of type IIa fibers significantly decreases after EE (55). Consistent with the present study results, both EE and RE have a beneficial effect on the peripheral skeletal muscle mass of patients with COPD, and EE seems to bring more changes in the aerobic metabolism phenotype. The exercise intervention mechanism to improve COPD skeletal muscle mass may be related to inhibiting the level of systemic inflammation, promoting skeletal muscle protein synthesis, muscle hypertrophy and regeneration, and improving the skeletal muscle metabolic enzyme activity (56).

Although there was a high heterogeneity in the methods and programs used to assess muscle strength, the results of this study still confirmed the significant positive effect of exercise on improving peripheral skeletal muscle strength in stable COPD. Subgroup analysis for different exercise modalities found that RE showed significant effects. We speculated that RE was designed for specific muscle groups that have less pressure on ventilation load and can effectively improve neuromuscular adaptation (57). Previous studies hypothesized that high-intensity whole/local body EE is sufficient to induce changes in the morphology and function of peripheral skeletal muscles in COPD (14). In the present study, only Alcazar et al. applied a 12-week high-intensity interval training program (high intensity: 80–90% *W*_peak_ and low intensity: 40–50% *W*_peak_) in stable COPD patients and found that the maximum isometric contraction strength and the force development rate of leg press significantly improved (21). Hence, the dose-response relationship between EE intensity and effect still needs to be determined. Also, there was a high heterogeneity in the pooled estimates of CE, and the heterogeneity decreased after a sensitivity analysis excluding the results from van Wetering et al., but still without reaching statistical significance. In the analysis of the literature characteristics, we found that the quadriceps muscle strength of the participants was 92–95% of the normal predicted value (47), which may lead to a small potential for improvement. However, the results are still inconsistent with speculations and previous research results, that is, CE has similar or even greater effects than EE and RE alone (16, 17, 40), which may be attributed to a variety of CE programs included in this meta-analysis. First, the proportion of EE and RE in CE programs. Most programs scheduled EE and RE in one session and two to three sessions a week, respectively, apart from the program in Zambom-Ferraresi et al. (scheduled EE in one session and RE in another session, only two sessions a week). Second, the range of exercise intensity was relatively extensive, which may play a role in maintenance but not in improvement. Therefore, in the CE program for improving COPD's skeletal muscle strength, the different proportions and intensities of EE and RE might have different effects, and it is still necessary to explore the best program.

Subgroup analysis for different muscle strength testing methods found that exercise can only significantly improve isotonic muscle strength. We speculated that the isotonic muscle strength test is more familiar to the participants and has a higher correlation with daily life than other tests (58). Considering that different strength units may be the source of heterogeneity, we pooled data units in kilograms and found that exercise significantly improved isometric muscle strength. Although the data of isokinetic muscle strength showed an increasing trend after exercise (33, 36), many studies are still needed to determine the degree of response. We also conducted subgroup analysis to determine the effects of exercise on upper limbs and lower limbs muscle strength and found that exercise can improve the muscle strength of both upper and lower limbs. Although subgroup analysis was performed, high heterogeneity still existed, and the source of heterogeneity was unclear. A standard and clinically feasible measurement program is needed to quantitatively evaluate the damage of peripheral skeletal muscle strength and the response to exercise in COPD.

Consistent with previous meta-analysis (15, 59), this study found that exercise can significantly improve 6MWD (26.64 m) in patients with COPD. However, only the EE improvement reached the minimal clinical important difference of 30 m (60), which may be attributed to EE bringing more aerobic metabolism changes and greater improvements in ventilation capacity; the relatively low proportion of EE in the CE program cannot bring significant improvement. The peak exercise capacity is often evaluated using a cardiopulmonary exercise test (CPET), which is considered the gold standard to assess the exercise capacity and closely related to COPD's prognosis (61, 62). A progressive incremental exercise protocol in a treadmill or cycle ergometer is often used for CPET, and the results can provide abundant physiological information related to exercise restriction, including the heart (e.g., heart rate, VO_2peak_, and oxygen pulse), lung (e.g., inspiratory capacity, gas exchange, and dynamic inflation), muscle (e.g., power and lactic acid), dyspnea (Borg), and exercise initiative (62). A Cochrane review conducted in 2015 showed that pulmonary rehabilitation (at least 4 weeks of exercise training) is beneficial in improving maximal exercise capacity (measured by *W*_max_) in patients with COPD, and the effect size exceeds the minimal clinically important difference (4 W) (63). Although a different outcome was used in this present study, the effect of exercise is confirmed. The comparison results of the effects of different modalities exercise showed no significant differences between RE vs. the control group (15, 64), RE vs. EE (16), and CE vs. EE (64) in improving the peak exercise capacity (VO_2peak_, *W*_peak_) of patients with COPD. It seems that a contradictory deduction might be concluded that exercise does not have a significant positive effect on peak exercise capacity of patients with COPD. Based on the primary pathophysiological mechanisms of exercise limitation in patients with COPD undergoing CPET, including ventilatory abnormalities, pulmonary gas exchange abnormalities, and skeletal muscle dysfunction (61), exercise with different modalities seems beneficial in improving peak exercise capacity in patients with COPD. Consistent with the hypothesis, this meta-analysis showed that exercise could significantly improve COPD's peak exercise capacity (1.82 ml/kg/min), and both EE and CE have positive effects.

This systematic review and meta-analysis had some limitations. First, there were flaws in methodological quality of the original studies, namely the lack of subject blinding and evaluator blinding in exercise intervention trials. Second, one of the included literatures had an apparently large sample size, which may have had an impact on the research results. Sensitivity analysis was performed to reduce the impact when high heterogeneity was found. Third, we only analyzed the effects of exercise on skeletal muscle strength and still needed to explore the effects of exercise on skeletal muscle endurance and power. Fourth, the outcomes of skeletal muscle function were not assessed comprehensively in most of the included studies, which may cause a limitation. Fifth, trial designs were heterogeneous. For high heterogeneity, we used a random-effects model and subgroup analysis to analyse the source of heterogeneity, and the results were consistent.

## Conclusions

**Figure 6:**
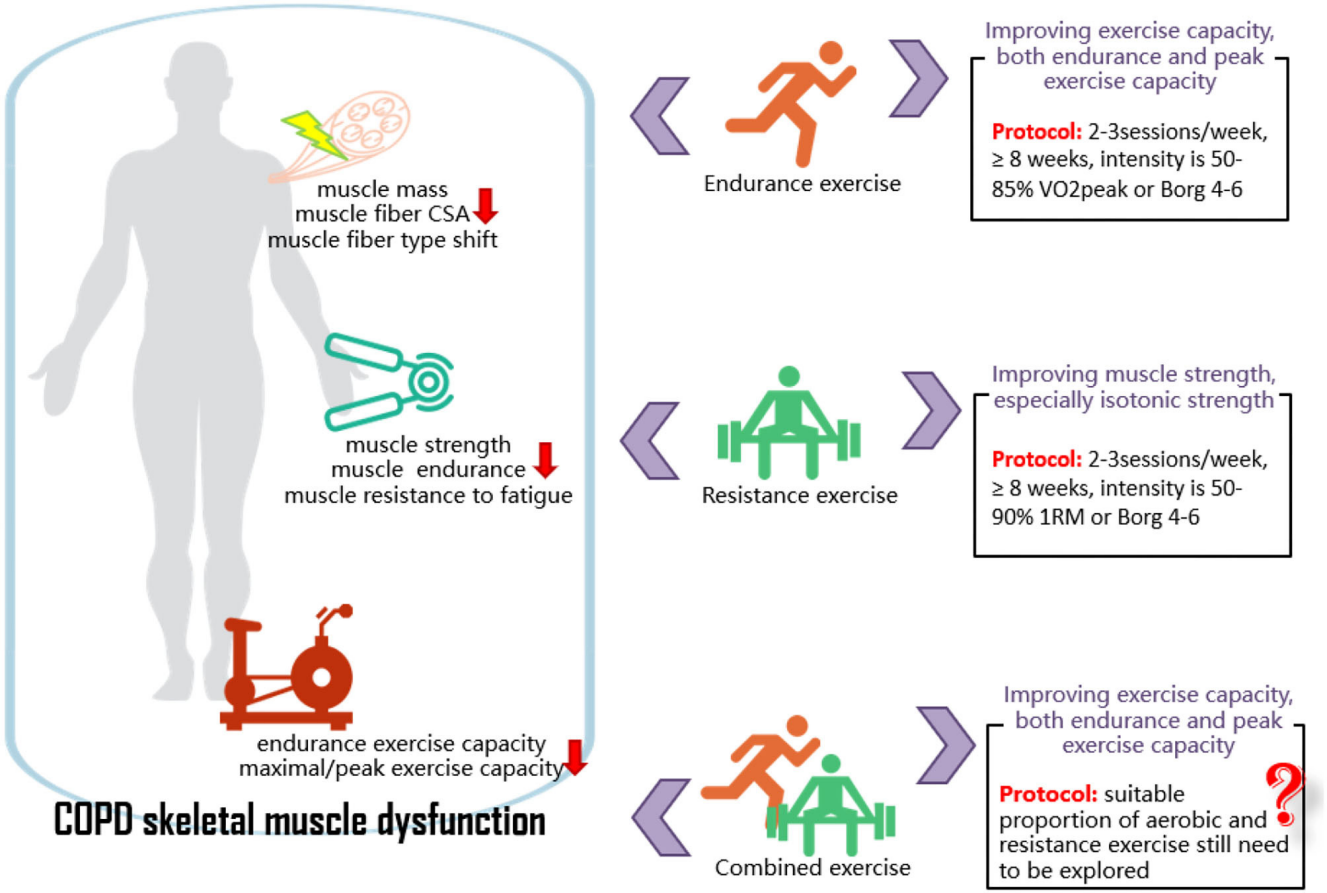
COPD skeletal muscle dysfunction and the effects of exercise on it. COPD, chronic obstructive pulmonary disease; CSA, cross-sectional area; RM, repetition maximum; VO_2peak_, peak oxygen consumption.

Exercise with different modalities seems effective in improving peripheral skeletal muscle strength and exercise capacity in patients with stable COPD. Specifically, EE shows a greater improvement in endurance and peak exercise capacity, and RE shows a greater improvement in peripheral skeletal muscle strength, and the isotonic test is relatively sensitive in reflecting muscle strength changes. Therefore, for patients with COPD whose exercise limitation is caused by a decreased cardiorespiratory capacity, EE might be a suitable choice. EE can be conducted in cycling, running, and walking, with an intensity of 50–85% VO_2peak_, 2–3 times/week, for at least 8 weeks. For patients with COPD whose exercise limitation is caused by an impaired peripheral skeletal muscle function, RE might be a preferable intervention. RE can be conducted in weight machines, free weights, and elastic bands, with an intensity of 50–90% 1RM, 2–3 times/week, for at least 8 weeks. The proportion of EE and RE in CE programs still needs to be explored and analyzed (Figure 6). High methodological quality RCTs with a large sample size are still needed to verify the present study results because of the relatively small inclusion of literature on the peripheral skeletal muscle structure and function in patients with COPD. It is also necessary to explore the effect of exercise intervention on peripheral skeletal muscle in AECOPD or patients with COPD with different severity.

## Data Availability Statement

The original contributions presented in the study are included in the article/Supplementary Material, further inquiries can be directed to the corresponding author/s.

## Author Contributions

XL and JX conceived of the idea for this review. JL and YW did the literature search. PL and YW collected the data. PL and JL did the quality assessment. PL did the statistical analyses and wrote the first draft of the manuscript. All authors analyzed and interpreted the data and revised and approved the final manuscript for submission.

## Funding

This study was funded by the National Natural Science Foundation of China, grant numbers 81902307 and 82072551. The funder of the study played no role in the study design, data collection, data analysis, data interpretation, or writing of the report.

## Conflict of Interest

The authors declare that the research was conducted in the absence of any commercial or financial relationships that could be construed as a potential conflict of interest.

## Publisher's Note

All claims expressed in this article are solely those of the authors and do not necessarily represent those of their affiliated organizations, or those of the publisher, the editors and the reviewers. Any product that may be evaluated in this article, or claim that may be made by its manufacturer, is not guaranteed or endorsed by the publisher.

## Abbreviations

6MWD: 6-min walking distance
ATS/ERS: the American Thoracic Society/European Respiratory Society
BMI: body mass index
CE: combined aerobic and resistance exercise
COPD: chronic obstructive pulmonary disease
CPET: cardiopulmonary exercise test
CSA: cross-sectional area
EE: endurance exercise
FEV_1_: forced expiratory volume in 1 s
FFM: fat-free mass index
GOLD: Global strategy for the diagnosis, management, and prevention of chronic obstructive pulmonary disease
GRADE: the Grading of Recommendations Assessment, Development and Evaluation
MD: mean difference
MeSH: medical subject headings
OR: odds ratio
PEDro: the Physiotherapy Evidence Database
PRISMA: the Preferred Reporting Items for Systematic Reviews and Meta-Analysis
RE: resistance exercise
SMD: standardized mean difference
VO_2peak_: peak oxygen consumption.
