# Supplementary material for: Effects of Exercise Intervention on Peripheral Skeletal Muscle in Stable Patients With COPD: A Systematic Review and Meta-Analysis
Source: Front Med (Lausanne). 2021 Nov 18;8:766841. doi: 10.3389/fmed.2021.766841 (PMC8636927; doi:10.3389/fmed.2021.766841)
Supplement: Supplementary file 1 [file Table_1.docx]

**Table S1 PEDro scale item score summaries**

| **Study/Year** | **Eligibility criteria** | **Random allocation** | **Concealed allocation** | **Baseline comparability** | **Blind subjects** | **Blind therapists** | **Blind assessors** | **Adequate follow-up** | **Intention-to-treat analysis** | **Between-group comparisons** | **Point estimates** | **Total score** |
| --- | --- | --- | --- | --- | --- | --- | --- | --- | --- | --- | --- | --- |
| Alcazar2019^37^ | Yes | 1 | 0 | 1 | 0 | 0 | 0 | 0 | 0 | 1 | 1 | 4 |
| Barakat2008^33^ | Yes | 1 | 0 | 1 | 0 | 0 | 1 | 1 | 0 | 1 | 1 | 6 |
| Borghi-silva2009^42^ | Yes | 1 | 0 | 1 | 0 | 0 | 0 | 0 | 0 | 1 | 1 | 4 |
| Borghi-silva2015^43^ | Yes | 1 | 1 | 1 | 0 | 0 | 0 | 0 | 0 | 1 | 1 | 5 |
| Cameron-tucker2014^24^ | Yes | 1 | 1 | 1 | 0 | 0 | 0 | 0 | 1 | 1 | 1 | 6 |
| Casaburi2004^28^ | Yes | 1 | 0 | 1 | 0 | 0 | 0 | 1 | 0 | 1 | 1 | 5 |
| Clark2000^38^ | Yes | 1 | 0 | 1 | 0 | 0 | 0 | 1 | 0 | 1 | 1 | 5 |
| Chen2018^35^ | Yes | 1 | 0 | 1 | 0 | 0 | 0 | 1 | 1 | 1 | 1 | 6 |
| de Souto Araujo2012^44^ | Yes | 1 | 0 | 1 | 0 | 0 | 0 | 0 | 0 | 1 | 1 | 4 |
| Emery1998^26^ | Yes | 1 | 1 | 1 | 0 | 0 | 0 | 1 | 0 | 1 | 1 | 6 |
| Gallo-silva2019^45^ | Yes | 1 | 1 | 1 | 0 | 0 | 0 | 0 | 0 | 1 | 1 | 5 |
| Hoff2007^36^ | Yes | 1 | 0 | 1 | 0 | 0 | 0 | 1 | 1 | 1 | 1 | 6 |
| Janaudis-ferreira2011^29^ | Yes | 1 | 1 | 1 | 1 | 0 | 1 | 1 | 1 | 1 | 1 | 9 |
| Lahham2020^47^ | Yes | 1 | 1 | 1 | 0 | 0 | 1 | 1 | 1 | 1 | 1 | 8 |
| Mehri2007^21^ | Yes | 1 | 0 | 1 | 0 | 0 | 0 | 1 | 0 | 1 | 1 | 5 |
| Mendes2010^48^ | Yes | 1 | 0 | 1 | 0 | 0 | 0 | 0 | 0 | 1 | 1 | 4 |
| Nakamura2008^31^ | Yes | 1 | 0 | 1 | 0 | 0 | 0 | 1 | 0 | 1 | 1 | 5 |
| Nyberg2015^39^ | Yes | 1 | 1 | 1 | 0 | 0 | 1 | 1 | 1 | 1 | 1 | 8 |
| O’shea2007^40^ | Yes | 1 | 1 | 1 | 0 | 0 | 1 | 0 | 1 | 1 | 1 | 7 |
| Petersen2008^50^ | Yes | 1 | 0 | 1 | 0 | 0 | 0 | 0 | 1 | 1 | 1 | 5 |
| Pradella2015^46^ | Yes | 1 | 0 | 1 | 0 | 0 | 0 | 1 | 0 | 1 | 1 | 5 |
| Simpson1992^25^ | Yes | 1 | 0 | 1 | 0 | 0 | 1 | 1 | 0 | 1 | 1 | 6 |
| Thabitha2012^23^ | Yes | 1 | 0 | 0 | 0 | 0 | 0 | 1 | 0 | 1 | 1 | 4 |
| Tsai2017^49^ | Yes | 1 | 1 | 1 | 0 | 0 | 1 | 1 | 1 | 1 | 1 | 8 |
| van Wetering2010^34^ | Yes | 1 | 1 | 1 | 0 | 0 | 1 | 1 | 1 | 1 | 1 | 8 |
| Wadell2004^27^ | Yes | 1 | 0 | 1 | 0 | 0 | 0 | 1 | 1 | 1 | 1 | 6 |
| Wadell2013^41^ | Yes | 1 | 0 | 1 | 0 | 0 | 0 | 1 | 0 | 1 | 1 | 5 |
| Weiner2000^32^ | Yes | 1 | 0 | 1 | 0 | 0 | 0 | 1 | 0 | 1 | 1 | 5 |
| Wiyono2006^22^ | Yes | 1 | 0 | 1 | 0 | 0 | 0 | 1 | 0 | 1 | 1 | 5 |
| Zambom-ferraresi2015^30^ | Yes | 1 | 0 | 1 | 0 | 0 | 1 | 1 | 1 | 1 | 1 | 7 |

**Notes:** Item “Eligibility criteria” does not contribute to total score. 1 = meets criteria, 0 = does not meet criteria.

**Table S2** Reasons of attrition in exercise and control groups

| **Author/Year** | **EG/CG attrition number** | **EG/CG attrition reasons** |
| --- | --- | --- |
| Alcazar2019^37^ | 5/1 | 2 transport issues, 2 non-study-related exacerbation, 1 non-study-related exacerbation heart shock/  1 non-study-related exacerbation |
| Barakat2008^33^ | 5/4 | 3 had an association with sleep apnea syndrome, 2 absence for 4 consecutive sessions/  2 had an association with sleep apnea syndrome, 2 had a cardiac problem during the first month of the program |
| Borghi-silva2009^42^ | 0/6 | /3 AE, 1 died, 1 newly diagnosed ischemic heart disease, 1 psychiatric problem |
| Borghi-silva2015^43^ | 7/5 | 3 refused to continue, 4 non-physiological cardiovascular response in maximal exercise test/  2 refused to continue, 3 non-physiological cardiovascular response in maximal exercise test |
| Cameron-tucker2014^24^ | 5/10 | 3 unwell, 2 others/  3 unwell, 4 family or social issues or appointments, 3 others |
| Casaburi2004^28^ | 1/1 | Both are non-protocol-related health problems |
| Clark2000^38^ | / | / |
| Chen2018^35^ | 4/4 | 1 travel abroad, 1 acute ileus, 1 cataract surgery, 1 abrupt dizziness/  1 cataract surgery, 3 not so serious |
| de Souto Araujo2012^44^ | 1/3 | 1 AE/  1 AE, 2 not completed all tests |
| Emery1998^26^ | 4/2 | 4 illness/ 2 transportation problems |
| Gallo-silva2019^45^ | 2/3 | All AE |
| Hoff2007^36^ | 0/0 | 0 |
| Janaudis-ferreira2011^29^ | 4/1 | 2 medical reason unrelated to the study, 1 did not like to train on the multi-gym, 1 AE unrelated to the study/  1 medical reason unrelated to the study |
| Lahham2020^47^ | 3/4 | All decline 6MWD test (questionnaires only) |
| Mehri2007^21^ | 0/0 | 0 |
| Mendes2010^48^ | 23/0 | 19 abandoned program, 4 lost to follow up |
| Nakamura2008^31^ | / | / |
| Nyberg2015^39^ | 2/2 | 2 AE/ 1 AE, 1 disappointed with group allocation |
| O’shea2007^40^ | 7/3 | 3 AE, 1 illness unrelated to COPD, 2injury unrelated to the trial, 1 family issues/  1 AE, 1 illness unrelated to COPD, 1 family issues |
| Petersen2008^50^ | 0/4 | /4 scheduling conflicts or the burden of the procedures |
| Pradella2015^46^ | 3/3 | 1 died, 1 AE, 1 withdraw consent/ 1 AE, 2 withdraw their consent |
| Simpson1992^25^ | 3/3 | 1 chest infection, 2 changes in their treatment/ 3 failed to return |
| Thabitha2012^23^ | / | / |
| Tsai2017^49^ | 1/0 | 1 died unrelated to the study |
| van Wetering2010^34^ | 15/9 | 7 withdraw their consent, 4 co-morbidity, 2 illness of family number, 2 died/  6 withdraw their consent, 3 co-morbidity |
| Wadell2004^27^ | 1/1 | Both failed to return |
| Wadell2013^41^ | 3/4 | 2 AE, 1 non-compliant with the exercise program due to knee problems/ 3 AE, 1 symptomatic cardiac arrhythmia |
| Weiner2000^32^ | 1/1 | Both lack of compliance |
| Wiyono2006^22^ | 3/1 | 3 refuse to continue the exercise/ 1 hospitalized due to uncontrolled diabetes mellitus type 2 |
| Zambom-ferraresi2015^30^ | 1/2/1 | All personal complications |

6MWD, 6-min walking distance; AE, acute exacerbation of COPD; CG, control group; EG, exercise group.

/ Not accessible
