# Supplementary material for: Effects of Exercise Intervention on Peripheral Skeletal Muscle in Stable Patients With COPD: A Systematic Review and Meta-Analysis
Source: Front Med (Lausanne). 2021 Nov 18;8:766841. doi: 10.3389/fmed.2021.766841 (PMC8636927; doi:10.3389/fmed.2021.766841)
Supplement: Supplementary file 2 [file Data_Sheet_1.pdf]

Study or Subgroup	Exercise Events	Exercise Total	Control Events	Control Total	Weight	Odds Ratio M-H, Random, 95% CI
**4.1.1 Endurance exercise versus control**						
Alcazar 2019	5	14	1	15	2.7%	7.78 [0.78, 77.93]
Barakat 2008	5	40	4	40	6.3%	1.29 [0.32, 5.19]
Borghi-silva 2009	0	20	6	20	1.7%	0.05 [0.00, 1.04]
Borghi-silva 2015	7	17	5	15	6.0%	1.40 [0.33, 5.93]
de Sauto Araujo 2012	1	14	3	14	2.5%	0.28 [0.03, 3.11]
Gallo-silva 2019	2	12	3	12	3.5%	0.60 [0.08, 4.45]
Mehri 2007	0	20	0	18		Not estimable
Petersen 2008	0	9	4	14	1.6%	0.12 [0.01, 2.59]
Pradella 2015	3	32	3	18	4.5%	0.52 [0.09, 2.88]
Wiyono 2006	3	30	1	31	2.7%	3.33 [0.33, 33.99]
**Subtotal (95% CI)**		**208**		**197**	**31.5%**	**0.84 [0.37, 1.93]**
Total events	26		30			
Heterogeneity: Tau² = 0.50; Chi² = 11.88, df = 8 (P = 0.16); I² = 33%						
Test for overall effect: Z = 0.40 (P = 0.69)						
**4.1.2 Resistance exercise versus control**						
Casaburi 2004	1	13	1	13	1.8%	1.00 [0.06, 17.90]
Chen 2018	4	29	4	26	5.6%	0.88 [0.20, 3.94]
Clark 2000	0	26	0	17		Not estimable
Hoff 2007	0	6	0	6		Not estimable
Janaudis-ferreira 2011	4	17	1	19	2.7%	5.54 [0.55, 55.49]
Nyberg 2015	2	22	2	22	3.3%	1.00 [0.13, 7.81]
Oshea 2007	7	27	3	27	5.8%	2.80 [0.64, 12.26]
Simpson 1992	3	17	3	17	4.3%	1.00 [0.17, 5.83]
Thabitha 2012	0	0	0	0		Not estimable
Zambom-ferraresi 2015	1	15	1	9	1.8%	0.57 [0.03, 10.43]
**Subtotal (95% CI)**		**172**		**156**	**25.3%**	**1.43 [0.69, 2.95]**
Total events	22		15			
Heterogeneity: Tau² = 0.00; Chi² = 3.25, df = 6 (P = 0.78); I² = 0%						
Test for overall effect: Z = 0.96 (P = 0.34)						
**4.1.3 Combined exercise versus control**						
Cameron-tucker 2014	5	43	10	41	8.2%	0.41 [0.13, 1.32]
Emery 1998	4	30	2	24	4.2%	1.69 [0.28, 10.13]
Lahham 2020	3	29	4	29	5.1%	0.72 [0.15, 3.55]
Mendes 2010	23	46	0	29	1.8%	59.00 [3.40, 1023.13]
Nakamura 2008	0	10	0	10		Not estimable
Tsai 2017	1	20	0	17	1.4%	2.69 [0.10, 70.49]
van Wetering 2010	15	102	9	97	11.9%	1.69 [0.70, 4.06]
Wadell 2004	1	15	1	13	1.8%	0.86 [0.05, 15.22]
Wadell 2013	3	17	4	24	4.9%	1.07 [0.21, 5.55]
Weiner 2000	1	18	1	5	1.7%	0.24 [0.01, 4.62]
Zambom-ferraresi 2015	2	16	1	9	2.2%	1.14 [0.09, 14.68]
**Subtotal (95% CI)**		**346**		**298**	**43.2%**	**1.19 [0.57, 2.50]**
Total events	58		32			
Heterogeneity: Tau² = 0.48; Chi² = 14.55, df = 9 (P = 0.10); I² = 38%						
Test for overall effect: Z = 0.46 (P = 0.64)						
**Total (95% CI)**		**726**		**651**	**100.0%**	**1.12 [0.75, 1.67]**
Total events	106		77			
Heterogeneity: Tau² = 0.15; Chi² = 29.24, df = 25 (P = 0.25); I² = 15%						
Test for overall effect: Z = 0.55 (P = 0.58)						
Test for subgroup differences: Chi² = 0.89, df = 2 (P = 0.64), I² = 0%						

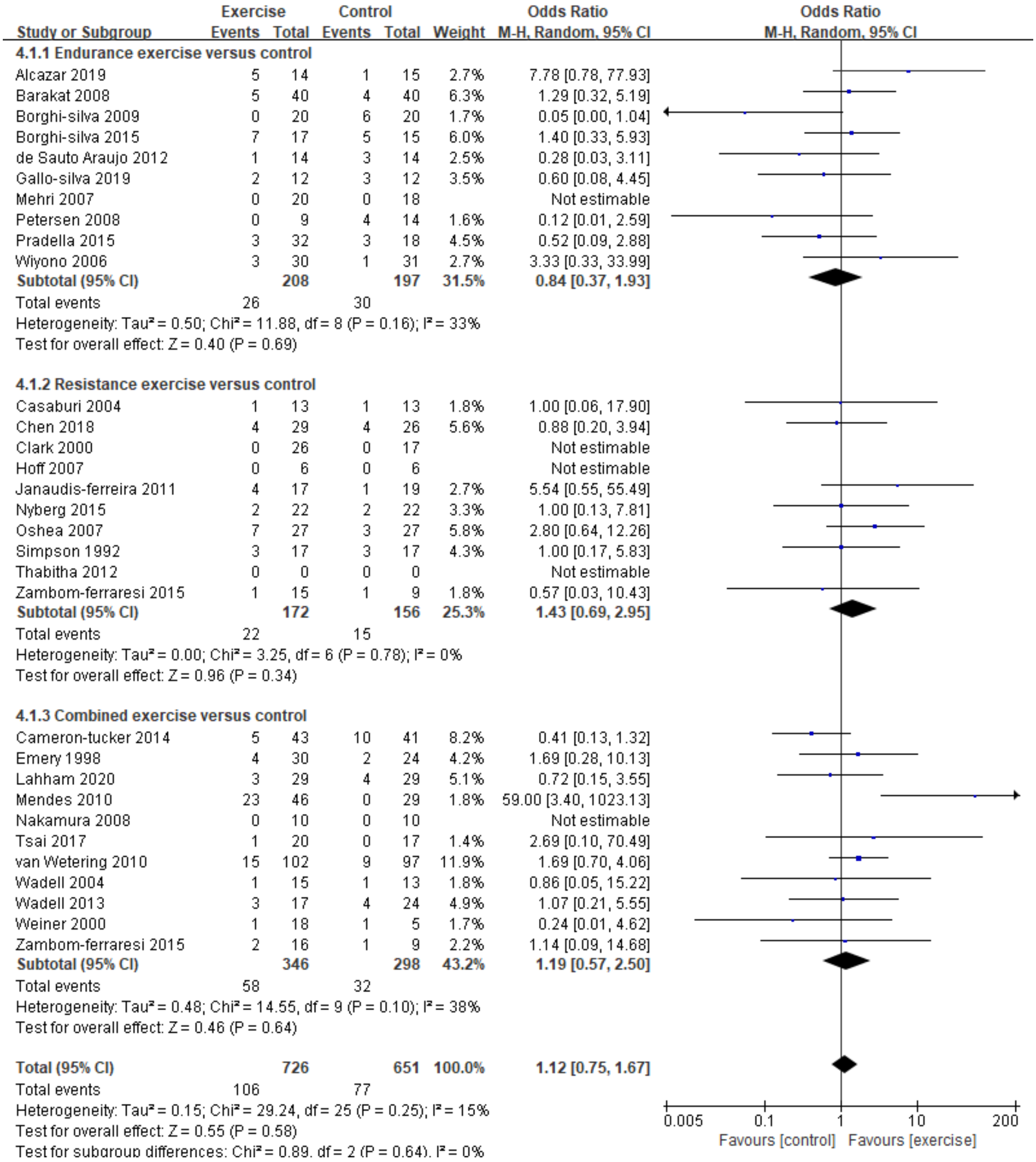

Figure S1 Pooled effect of exercise compared with control on attrition number.
CI, confidence interval; OR, odds ratio; SD, standard deviation.

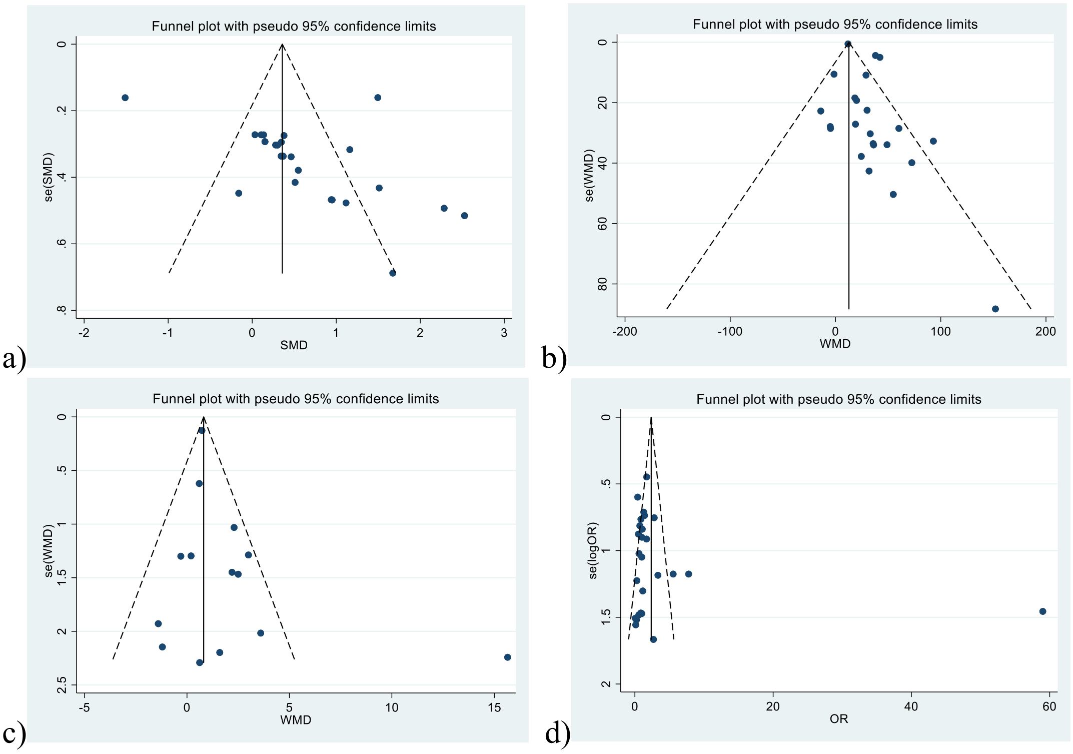

Figure S2 Funnel plot showing SE and effect size in trials of exercise for a) skeletal muscle strength, b) 6MWD, c) VO_2 peak, d) attrition number in COPD patients.

6MWD, 6-min walking distance; COPD, chronic obstructive pulmonary disease; OR, odds ratio; SMD, standardized mean difference; VO_2 peak, peak oxygen consumption; WMD, weighted mean difference.

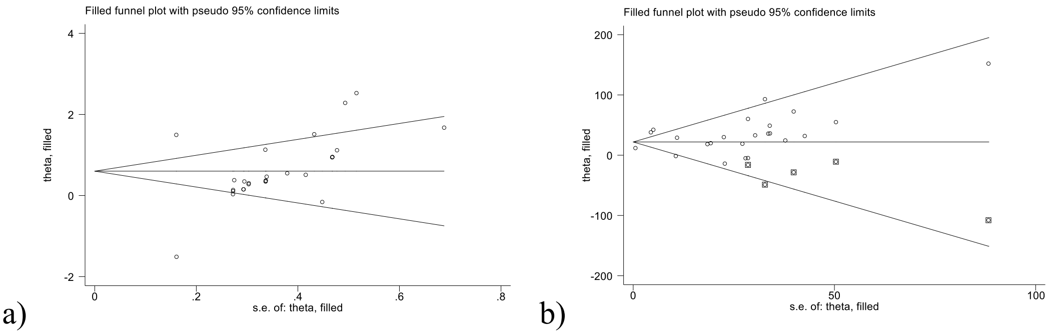

Figure S3 Trim and fill methods showing the filled studies need to diminish the publication bias (the circles enclosed in boxes are filled studies). a) skeletal muscle strength, b) 6MWD.

6MWT, 6-min walking distance.
